# Prescribing errors by junior doctors- A comparison of errors with high risk medicines and non-high risk medicines

**DOI:** 10.1371/journal.pone.0211270

**Published:** 2019-01-31

**Authors:** Mahdi A. Alanazi, Mary P. Tully, Penny J. Lewis

**Affiliations:** Division of Pharmacy & Optometry, School of Health Sciences, Faculty of Biology, Medicine and Health, University of Manchester, Manchester, United Kingdom; Macquarie University, AUSTRALIA

## Abstract

**Introduction:**

Prescribing errors in hospital are common. However, errors with high-risk-medicines (HRMs) have a greater propensity to cause harm compared to non-HRMs. We do not know if there are differences between the causes of errors with HRMs and non-HRMs but such knowledge might be useful in developing interventions to reduce errors and avoidable harm. Therefore, this study aims to compare and contrast junior doctors’ prescribing errors with HRMs to non-HRMs to establish any differences.

**Methods:**

A secondary analysis of fifty-nine interviews with foundation year doctors, obtained from three studies, was conducted. Using a Framework Analysis approach, through NVivo software, a detailed comparison was conducted between the unsafe acts, error-causing-conditions (ECCs), latent conditions, and types of errors related to prescribing errors with HRMs and non-HRMs.

**Results:**

In relation to unsafe acts, violations were described in the data with non-HRMs only. Differences in ECCs of HRMs and non-HRMs were identified and related to the complexity of prescribing HRMs, especially dosage calculations. There were also differences in the circumstances of communication failures: with HRMs ineffective communication arose with exchanges with individuals outside the immediate medical team while with non-HRMs these failures occurred with exchanges within that team. Differences were identified with the latent conditions: with non-HRMs there was a reluctance to seek seniors help and with HRMs latent conditions related to the organisational system such as the inclusion of trade names in hospital formularies. Moreover, prescribing during the on-call period was particularly challenging especially with HRMs.

**Conclusion:**

From this secondary analysis, differences in the nature and type of prescribing errors with HRMs and non-HRMs were identified, although further research is needed to investigate their prevalence. As errors with HRMs have the potential to cause great harm it may be appropriate to target limited resources towards interventions that tackle the underlying causes of such errors. Equally concerning, however, was the sense that doctors regard the prescribing of non-HRMs as ‘safe’.

## Introduction

Prescribing errors are said to occur when “*as a result of a prescribing decision or prescription writing process*, *there is an unintentional significant reduction in the probability of treatment being timely and effective or increase in the risk of harm when compared with generally accepted practice*”.[1] Such errors are the most frequent type of error in the medication use process[2, 3] and can double patients’ length of hospital stay and cost of care, as well as increasing their mortality rate.[4–6] With all types of medication, hospital prescribing errors are common[7] occurring with 7% of medication orders, 2% of patient days and 50% of hospital admission.[8] Seventy percent of hospital prescriptions are prescribed by Foundation Year 1 (FY1) and Foundation Year 2 (FY2) junior doctors.[9] Nonetheless they have twice the prescribing error rate compared to consultants.[10] Not all prescribing errors necessarily have the same consequences; about 41% are considered to be minor.[10] High Risk Medicines (HRMs) have a greater ability to cause devastating harm when they are used in error compared to regular medicines.

The UK’s National Health Service has defined HRMs as *“medicines that are most likely to cause significant harm to the patient*, *even when used as intended”*. [11] In the USA, the Institute for Safe Medication Practices (ISMP) uses the term high alert medication and defines them as *“drugs that bear a heightened risk of causing significant patient harm when they are used in error”*.[12] In the UK, the National Patient Safety Agency (NPSA) has produced its own list of HRMs which contains eight classes; anticoagulants, injectable sedatives, opiates, insulin, antibiotics (risk of allergy), chemotherapy, antipsychotics, and infusion fluids. [11]

Particularly worrying is that the prevalence of prescribing errors with HRMs is wide ranging and has been reported to reach as high as 89.6 errors per 100 orders of HRMs.[13] However, prescribing errors are preventable and obtaining a better understanding of their causes can lead to the development of strategies and techniques to reduce errors and hence reduce patient harm.

There have been many models used to categorise and classify the causes of prescribing errors. One of the most common is Reason’s model of accident causation [14]. This has been used to classify prescribing errors into different types of unsafe acts (or active failures) including knowledge based mistakes [15–20], rule based mistakes [21], slips and lapses [3, 17–19, 21], and violations [17, 18, 21–23]. The model also incorporates error-causing conditions (ECCs) which are conditions that predispose an individual to making an error and latent conditions that are distal to the error but can allow error causing conditions to manifest. These latter include management decisions and design decisions. Multiple ECCs and latent conditions are possible for a single prescribing error. This model of multifactorial influences was reflected in the findings of a systematic review about the causes of prescribing errors in hospitals.[24]. ECCs related to the emergence of prescribing errors have been described in previous studies and include the individual prescriber (e.g. physical health and knowledge) [3, 19, 21], working environment (e.g. staffing and accessibility for drug information) [3, 15, 16, 19–21, 25, 26], the healthcare team (e.g. poor communication and the relation with seniors) [16, 18, 19, 21, 22], prescribing task (e.g. non-routine tasks or protocols) [3, 19, 21] and the patient (e.g. difficulty with communication, complex disease) [19, 21]. Latent conditions described were varied and inconsistent but included factors such as reluctance to question greater authority [22], organizational system [25] and a lack of importance attached to prescribing [16, 19, 21].

These previous studies have all been based upon the investigation of prescribing errors made with all types of medication, without any distinction made between those errors made with non-HRMs and those made with HRMs. There may be many similarities, but there could also be important differences between these classes of medicines that may help in the development of interventions to reduce prescribing errors with HRMs. Considering the severe consequences of errors with these medicines, this could be an important focus for reducing actual harm from preventable adverse drug events.

However, to date the causes of prescribing errors with HRMs made by junior doctors has not been studied. Therefore, this study will focus on this area of potential high risk and fill the gap in this area of research.

### Aim

This study aims to compare and contrast the causes of junior doctors’ prescribing errors with HRMs with that of non-HRMs in order to establish any differences.

## Methods

### Design

This study used secondary analysis of existing data,[27] where the data were obtained from fifty-nine semi-structured interviews conducted by the research team in three previous studies of prescribing errors,[28–30] which were already audio recorded by the researchers then transcribed by professional transcribing company. The included data sets all related to studies that explored a directly related phenomenon, which provides some reassurance that the data would contain pertinent details relevant to our new research question relating to HRMs. Furthermore, the data collection approach of all three studies involved in-depth interviews from which it would be likely to extract relevant yet new information.[31] The secondary analysis of existing qualitative data, like interview data, has many advantages; for example, it provides a large amount of data that has been rigorously collected and provides a cost efficient way to address the research aim, avoiding the need for further data collection and transcribing.

The three previous studies on prescribing errors are summarised in Table 1. The recruitment process of these studies were started with approaching the participants by email, face-to-face or through the training tutors by providing an information sheet that described the purpose of the study prior to giving consent for interviews. The majority of participants, in Set 1 and Set 2, who agreed to participate, were purposely selected to represent a wide range of medical graduates from across the UK and working in different types of hospitals. In study 3, participants worked at a single hospital and were involved in a structured feedback intervention to improve antimicrobial prescribing. Five were in the intervention and five were in the control groups. All interviews in the three studies were conducted at the workplace of the participants to make it easy for them; therefore, participation rates were improved. However, there may have been responder bias which is inevitable in non-random sampling. Snowballing in both study 1 and 2 did, however, mean that a range of participants were recruited. All the studies had been granted ethical approval: North Manchester Research Ethics Committee, ref 07/H1006/93 (Set 1); Manchester University Research Ethics Committee, ref ethics/11345 (Set 2); Manchester University Research ethics Committee, ref ethics/12238 (Set 3). Consent was obtained from all participants for future analysis of their anonymised interview data.

**Table 1:** Summary of the three studies.

Set of interviews	Number of Participants	Interviewees*	Study information
**Set 1**	30	FY1 doctors (16 women, 14 men)	Interviews obtained from Lewis *et al* study[28] which discussed causes of prescribing errors in all medication types. The topic guide was divided into three main sections: Part 1: provided background about the participant such as place of medical education, speciality and time in post. Part 2: An in-depth exploration of prescribing errors participants had made previously including the nature of the error, situation of the error and participants reasons for making the error. Part 3: Participants’ experiences and attitudes towards basic medical education.
**Set 2**	19	FY2 doctors (13 women, 6 men)	Interviews obtained from a study conducted about prescribing errors in all medication types in FY2 doctors[29] The topic guide of this study was similar to set 1 with the exception of the part 3 of the schedule which instead explored the differences between FY1 and FY2 practice.
**Set 3**	10	FY1 doctors (6 women, 4 men)	Interviews obtained from McLellan *et al* study[30] about antimicrobial prescribing errors. The interviews explored the whole system of antimicrobial prescribing and the influence of both the intervention (structured feedback) and normal feedback practice. Questions and prompts covered prescribing practices and participants’ knowledge, skills, their beliefs on how they could change their practice.

* FY1 = Foundation Year 1, FY2 = Foundation Year 2.

The interviews from these studies were initially conducted to investigate the causes of prescribing errors in all medications types without distinguishing between HRMs and non-HRMs. As the interviewees were not asked about prescribing errors in relation to medications classification as HRMs or non-HRMs, participants had the opportunity to talk truthfully about the prescribing error without the influence of the term HRMs. Bias was also reduced as there was no previous relationship between the interviewers and the participants.

### Data analysis

An initial screening of all transcripts was undertaken by MA to ensure that each had information relevant to the aim of this study i.e. that prescribing errors were discussed, that it was possible to determine whether errors were related to HRM or non-HRMs and that discussions related to prescribing errors in the hospital setting. Medications were identified as HRMs according to both the ISMP[12] and NPSA lists of HRMs.[32]

Framework Analysis was used to analyse the data,[33] a common approach for health research.[34]This approach involved six stages; transcription, familiarisation, coding, developing an analytical framework, applying the framework, charting the data and interpretation.[35] NVivo software (version 10) was used to support this analysis.[36] The stages of analysis began with familiarization with the data by reading thoroughly. This was followed by inductive coding of the interviews, which gave better understanding of the data and allowed the researchers to draw key differences from the raw data under each medication type, HRMs and non-HRMs. The third step was deductive coding based on Reason’s model of accident causation. This model is described in detail below. The coding process was iterative; MA did the initial coding then this was discussed on multiple occasions with the other members of the research team (PL and MT) until a consensus was reached upon on an overall framework to apply to all transcripts. After this was complete, charting of the data allowed for a comprehensive and detailed comparison between HRMs and non-HRMs to distinguish any differences. To perform this robust comparison, a table was drawn to compare unsafe acts, ECCs, latent conditions and types of prescribing errors. The table of differences was iteratively developed by all researchers until it reached the final version. The final themes were the obvious differences that were associated to the specific drug class, HRMs or non-HRMs, with reasonable justification from the interviewees’ description or related to the nature of the drug class. The selected quotes were chosen based on their clear depiction of the highlighted theme. The quotes were referenced using the participant’s number and written in italic and bold font ***(****i*.*e***. *P#)***. Where extraneous material has been removed from the quote, this has been indicated using an ellipsis **(…)**

#### Reason’s model of accident causation

Reason’s model of accident causation, which is the most common model used to categorise the causes of prescribing error;[37] was used as part of the analytical framework to categorise the type of unsafe act, error-causing conditions, and the latent conditions resulting in prescribing errors.[14] Definitions of these terms are provided in Table 2. The types of unsafe acts were classified as knowledge and rule based mistakes, slips, lapses and violations; an additional category of communication errors was added based on our previous work.[28] Table 3 provides definitions of the unsafe act/active failure with examples from clinical practice. Some prescribing errors could be classified under more than one unsafe act and usually multiple ECC. In addition, the ECCs, for both HRM and non-HRMs, were classified under following categories: individual prescriber; healthcare team; prescribing task; the patient; and working environment.

**Table 2:** Definitions of unsafe acts, error causing conditions and latent conditions[14, 38].

**Unsafe acts (also termed active failures)**	These are the unsafe acts performed by people who are in direct contact with the patient or system. They are at the sharp end of the error. They are categorised into two main types; execution failures and planning failures. See Table 3 for more details.
**Error causing conditions (ECC)**	Unsafe acts or active failures are at the sharp end of errors but are not the sole causal factor. There are error-causing conditions that predispose an individual to making an error. These are also termed contributory factors and can relate to factors in the environment (e.g. workload), the task itself (e.g. complexity) the team (e.g. communication), individuals (e.g. knowledge) or the patient (e.g. complexity of medical condition).
**Latent conditions**	Described as inevitable “resident pathogens” within a system. They arise from decisions made by designers, builders, procedure writers and top level management. They are not a direct cause of errors but can translate into ECCs and introduce weaknesses in the defences allowing errors to manifest.

**Table 3:** Definitions and examples of the unsafe acts (active failures) of prescribing error (adapted [10, 14]).

Type of unsafe act	Definition
**1. Planning Failure**	Correct execution of inappropriate or incorrect plan
** a. Knowledge- Based Mistake**	Mistakes that occur at the knowledge based performance level. Occur when faced with a novel task and have to consciously construct a plan of action. Occur when an inappropriate plan or incorrect plan is correctly executed. Example: Not prescribing a loading dose of digoxin when initiating therapy, as did not know that this was required
** b. Ruled-Based Mistake**	Mistakes that occur at the rule-based performance level when drawing on a set of stored mental if-then rules. Occur when an inappropriate plan or incorrect plan is correctly executed. Example: Prescribing the wrong dose of enoxaparin, as did not take into account the patient’s renal function—just prescribed the regular dose as would normally do.
**2. Execution Failure**	Failure in the execution of a good plan
** a. Slips**	An error that is caused by a failure in performing an intended action and that is replaced by another action. Example: prescribing a medication on a discharge prescription in milligrams that should be prescribed in micrograms, as was writing out a list of other medications with doses in milligrams.
** b. Lapses**	An error that is caused by omission of a particular task. Example: Forgetting to check the drug chart to see if a patient is allergic to penicillin before prescribing an antibiotic.
**3. Violation**	An error that is caused by a conscious decision to ignore the accepted rules or procedures of the organization. Example: missing out necessary information on a prescription expecting pharmacy staff to complete it.
**4. Communication Errors**	An error that is caused by a lack of or an error in communication between the prescriber with the healthcare team, with other healthcare professionals, or with patients. Example: prescribing the wrong dose of a medication in hospital as the general practice staff provide erroneous information.

Prescribing errors were classified in five major types as adapted from a study by *Dean et al* [39] and applied in other studies of prescribing errors. [9, 40] The five major types of errors were ‘need for drug therapy’, ‘selection of a specific drug’, ‘selection of dosage regimen’, ‘administration of drug’, and ‘provide drug product’. Each major type of errors had its own subtypes of errors; for example, ‘need for drug therapy’ had subtypes such as omission errors, premature discontinuation, new drug not prescribed but indicated, continuation for longer than needed, a drug prescribed with no indication, and duplication.

## Results

Forty-seven out of the fifty-nine interviews were included in the analysis. The remaining 12 interviews were excluded because they did not mention any prescribing errors (n = 7), did not provide the name or type of medication in which the error occurred (n = 1), or because the prescribing error occurred outside hospital settings such as in general practice settings (n = 4). The 47 interviews contained descriptions of 108 prescribing errors; 39 errors with HRMs, for example, Low Molecular Weight Heparins (LMWH), insulin, narcotics and opioids, and antibiotic related allergy; and 69 with non-HRMs, for example, Non- Steroidal Anti- Inflammatory Drugs (NSAIDs), paracetamol, statins, and Proton Pump Inhibitors (PPIs).

### Differences in types of errors

The types of unsafe acts with HRMs included 10 knowledge based mistakes, 14 rule based mistakes, 10 lapses, 5 slips, and 5 communication errors (total no. = 44). The non-HRMs contained 17 knowledge based mistakes, 26 rule based mistakes, 9 lapses, 14 slips, 2 communication errors, and 6 violations (total no. = 74).

There were five major differences between the description of HRM errors and non-HRM errors (Table 4). From these five differences, two overarching themes emerged, one was in the types of unsafe act and the other was in the types of prescribing error. The first theme related to violations, which were an obvious difference between HRMs and non-HRMs as they were described by interviewees with non-HRMs only. The second theme related to the particular type of prescribing error ‘provide drug product’, which was described by interviewees with HRMs only.

**Table 4:** Differences in types of prescribing errors and violations between HRMs and non-HRMs.

	HRMs (n = 39)	Non-HRMs (n = 69)
**Type of unsafe act**		
**Violations**	Not described^(1)^	Described (n = 6)
**Types of prescribing errors**		
**Provide drug product**	KBM, RBM, Lapses	Violations
**Administration of drug**	KBM only	KBM, RBM, Lapses, Slips, Violations
	Subtype: • Wrong dosage form	Subtypes: • Wrong dosage form • Wrong dose timing • Wrong route • Wrong administration technique.
**Selection of specific drug**	KBM, RBM, CE, Lapses, Slips	KBM, RBM, Lapses, Slips
	Subtype: • Significant allergy	Subtypes: • Contraindication • Unintentional drug prescription
**Need for drug therapy**	RBM, CE, Lapses	KBM, RBM, Lapses, Slips, Violations
	Subtype: • Drug not prescribed	Subtypes: • Drug not prescribed • Drug duplication • Drug not needed
**Selection of dosage regimen**	Similar Except for violations as presented with non-HRMs only	

HRMs = High Risk Medicines, Non-HRMs = Non High Risk Medicines, KBM = Knowledge-Based Mistakes, RBMs = Ruled-Based Mistakes, CE = Communication Errors

^(1)^ = Underlined text to indicate where there is difference.

Violations as a type of unsafe act were one of the major differences between interviewees’ descriptions of HRMs and non-HRMs prescribing errors. Violations were described only with non-HRMs in four of the five types of prescribing errors (Table 4). The types of prescribing error included; ‘need for drug therapy’ which related to the premature discontinuation of medication; drug not prescribed but indicated and changing an accurate prescription to cover up another healthcare professional’s mistake; the ‘selection of dosage regimen’ which occurred when the prescriber did not check the dose frequency of a medication; the ‘administration of drug’ which related to omission of the medication stop date; and the ‘selection of dosage regimen’ which occurred when the prescriber wrote a wrong dose frequency without checking if the frequency was correct or not with the intention of obtaining the medication faster from the pharmacy.

One such error occurred where a prescriber did not write the antibiotic duration as he lacked the knowledge, the seniors’ support, and the resources to find the duration: ***“Sometimes if I don't know and I can't get hold of anyone and I can't get the BNF [British National Formulary] I don’t even write it [duration]*. *I don’t write for how many days*, *cos I know pharmacy will ring back*. *I’ve done that before*.*” (P5)***. Therefore, the prescriber violated the rules by intentionally sending the prescription to the pharmacy without duration. These were often routine violations, which occurred because the prescriber was in a hurry or had a high workload.

It is possible that prescribers choose to make these routine violations with non-HRMs because they know that these medications are less harmful to the patient when they are prescribed wrongly. In contrast, HRMs, by their definition, can have deleterious effects when prescribed erroneously. An example of this was described by an interviewee who knowingly prescribed nystatin incorrectly without checking the appropriate frequency, as he wanted to complete the task quickly. The prescriber believed it to be a ‘safe error’ as he felt that there would be little harm to the patient:

***“…it was*, *prescribing nystatin for a patient with oral candidiasis and I wasn’t sure of the dosing regimen*, *how many times a day to give it*, *and I put it down as*, *TDS [three times a day]… my thoughts were that the difference between TDS and QDS [four times a day] it's not a huge difference*, *it's not going to make*, *that much difference if I change it*, *if I checked BNF… so just to get it sent away I put it as sort of TDS… didn’t particularly have any feelings at that point cos I thought it was a*, *a safe*, *a safe error shall we say* …*”(P23)***.

Moreover, knowing that there was the existence of a safety net, such as senior colleagues or pharmacists (who would check the juniors’ prescriptions and correct them), together with the low risk of harm when prescribing non-HRMs, could lead to the belief that routine violations are acceptable behaviour. The junior prescribers relied on the safety net to fill gaps in their prescriptions or pick up on errors which would then be flagged along with the appropriate prescription instructions:

***“…they’re both long-term treatments rather than ones that are going to instantly help somebody*, *but it's just making sure I remember to do it*. *The good thing is that we do have*, *people to review those*, *and to sort of pick up on if they have or they haven’t been prescribed it…” (P12)***.

Another major difference between HRMs and non-HRMs was the frequency of the type of prescribing error “provide drug product”, which, in relation to HRMs, were mainly the legal requirements of controlled drug prescriptions. In the UK, controlled medications are HRMs and require additional details when prescribing, especially for discharge prescriptions, compared to prescriptions for other medications. Such errors were often lapses as the prescriber forgot to write the numbers also in words (a legal requirement in the UK for controlled medications), rule based mistakes where the prescriber did not apply the rules of controlled drug prescriptions because they did not realise the prescribed drug required it or knowledge based mistakes, where the prescriber lacked knowledge about how to complete the controlled drug prescription. An interviewee stated that:

***“Q***: ***And had you prescribed morphine before?***

***A: I'd prescribed it on a normal prescription chart, but not as, a take-home***.

***Q***: ***And are they prescribed differently?***

***A***: ***Yeah…***

***Q***: ***And why do you think you made the error?***

***A: I'd just never been taught how to do it and, and then we since, just shortly after that, in fact, we had a teaching session…” (P18)***.

All the errors with HRMs that were classified under ‘provide drug product’ occurred with discharge medication prescriptions for controlled drugs. This transition period of time, when a patient is discharged from hospital, can be vulnerable to prescribing errors as prescribers often have a high workload requiring them to multi-task under time pressure. One interviewee described the situation when he made this error during the discharge period time:

***“… I think a lot of it is because you are so busy and half the time you’re doing two, three things at once. Like, I can say, there’s been many occasions like I’d be on the phone trying to sort something out and writing a TTO [discharge medication] at the same time, and you really shouldn’t do that.” (P13)***.

### Differences in error causing conditions

There were similarities and differences in the ECCs of HRMs and non-HRMs errors (Table 5). Two key themes were drawn from these differences between HRMs and non-HRMs. The first theme related to the complexity of the task of prescribing HRMs and the second was related to the nature of communication failures.

**Table 5:** Differences in Error Causing Conditions (ECCs) between HRMs and non-HRMs.

ECC	HRMs	Non-HRMs
**Individual prescriber**		Acting in an automatic way^(1)^ • Slips
**Healthcare team**		Lack of senior support • RBMs, Slips
	Communication failure outside immediate medical team • Communication errors	Communication failure inside immediate medical team • Communication errors
**Prescribing task**	Complicated task • KBM, RBM	
**The patient**	Similar (e.g. complexity of medical condition, severity of medical condition)	
**Working environment**	Similar (e.g. busyness, workload)	

ECCs = Error Causing Conditions, HRMs = High Risk Medicines, Non-HRMs = Non High Risk Medicines, KBM = Knowledge-Based Mistakes, RBMs = Ruled-Based Mistakes.

^(1)^ = Underlined text to indicate where there is difference.

When comparing HRMs to non-HRMs, the nature of the prescribing task was different in two ways–the occurrence of knowledge based and rule based mistakes. These differences represented some traits of prescribing HRMs in general, as some can be complicated to prescribe or can be infrequently prescribed. Participant 24, as an example, described the rule based mistake of prescribing dalteparin for a patient with a deteriorated renal function and the complexity of the task for him, and said: ***“…it was the whole ideal body weight thing and working out eGFR [estimated Glomerular Filtration Rate] cos essentially*, *eGFR I was fine*, *GFR was deranged and just still don’t really don’t know how to work out GFR*, *it’s so complicated*.*” (P24)*.**

The complicated tasks described were particularly associated with calculations and prescribers’ difficulty in completing these calculations. Such calculations require intensive training and the provision of calculation instructions over the phone would not help much in understanding of these tasks. Interviewees described a lack of calculation training or instruction resulting in mistakes, one interviewee stated:

***“Q: Okay. And who had asked you to prescribe those?***

***A***: ***The GU [genitourinary] Consultant, over the phone.***

***Q***: ***Okay. And did you ask anybody about this?***

***A: Well I asked him. And he was, like because, oh Microbiology also told me, because he [the patient] was quite poorly. So I had them both ringing me over the phone… But it was really complicated… The whole process that I wrote out for them was fine. But it was just the actual dose, because of the dilution, or the fact on how many grams per whatever.” (P16)***.

Another interviewee described their vague recollection of calculation training in relation to anticoagulant dosing: “…***I didn’t know how much to give her and I think it’s just as part of our training we need to know about well I possibly did have a tutorial once on ideal body weight but it probably needs to be highlighted in our training*.*” (P24)*.**

Communication errors occurred with both HRMs and non-HRMs. However, there was a difference between them. Communication failures with non-HRMs arose within the immediate medical team, i.e. between a junior and a senior prescriber within the same team. Seniors were described as not providing enough details or proper instructions for the juniors. For example, one interviewee prescribed the wrong infusion rate of omeprazole after basing his decision on incorrect instructions from a senior doctor: ***“…I was just confused because nobody actually told me how to do it*, *so I was just verbally taught it*, *informally taught and I just prescribed it but it was detected by the pharmacist basically*.*” (P25)*.** Moreover, the junior doctor did not check the information that had been given by their senior:

***“Q***: ***And had you looked it up or?***

***A: No, basically the registrar just said yeah, this is how it is.” (P25)***.

Both senior and junior doctors did not give their full attention to the prescribing task, which could be related to the relatively good safety profile of non-HRMs.

In contrast, none of the communication failures with HRMs in this study occurred as part of this “team prescribing”. The problems were with individuals outside of the medical team such as communication failures between the prescriber and the patient, the GP, or another healthcare professional. This could be related to the high awareness within the medical team to the importance of these HRMs and the consequences of committing a mistake; therefore, communications regarding them may be clearer than with non-HRMs.

There were other, less notable differences such as an individual prescriber acting in automatic way with non-HRMs, as one interviewee stated when he prescribed a laxative (Senna) instead of potassium supplement (Sando K): ***“I think I might have gone back to being automatic*.*” (P39)***, but this kind of difference could occur with HRMs as well; therefore, not every difference was included as a theme. There were some ECCs that were, described many times by the interviewees with both HRMs and non-HRMs as they were challenging for the prescribing task (i.e. on-call period and TTO). However, the on-call period was more often described with HRMs than non-HRMs, one participant described the on-call period as ‘crazy’ as he states:

***“Q***: ***About this particular example, was it especially busy or was it just like a normal on-call?***

***A***: ***There’s no such thing as especially busy on call they’re all the same…***

***Q***: ***Is it?***

***A: Yeah they’re all busy, they’re all crazy.”(P27)***.

### Differences in latent conditions

Differences in latent conditions were found with mistakes and violations with HRM and non-HRMs (Table 6). However, as well as in the unsafe acts, types of prescribing errors and ECC not all the differences could be transferred to themes. Therefore, two themes were identified: reluctance to ask seniors was a latent condition described only with the knowledge and rule based mistakes occurring with non-HRMs and ‘organisation documents leading to error’ which was found only in knowledge based mistakes with HRMs.

**Table 6:** Differences in latent conditions between HRMs and non-HRMs.

Latent condition	Types of unsafe act	
	HRMs	Non-HRMs
**Organisation documents contribute to error**	KBM	
**Previous decision made by seniors**	RBM	
**Prescriber reluctant to ask seniors**		KBM, RBM
**Poor staff organisation**		KBM
**Shortage of resources**		Violation

HRMs = High Risk Medicines, Non-HRMs = Non High Risk Medicines, KBM = Knowledge-Based Mistakes, RBMs = Ruled-Based Mistakes

A reluctance to ask seniors or to look up drug information in front of other healthcare professionals was a latent condition that was only described with knowledge based mistakes in non-HRMs. Junior doctors felt they would be perceived as incompetent, stupid, or that they would annoy seniors if they asked or looked up information about such drugs. An example of this was given by an interviewee when he prescribed the wrong dose of ibuprofen, as said:

***“…someone had asked me to prescribe it and I didn’t want to look like I had to look it up***.

***Q: Okay***.

***A: Cos, you know, I didn’t want to look stupid, so I, I just, I was like, I was thinking, “Yeah, I'm pretty sure it's not a round number, like 500 or 1000, I think it's about 300/400,” so I just put 300” (P18)***.

The medication type that was being prescribed played a role in prescriber reluctance to ask seniors for advice or help. Those medications that were perceived as being simple, and were therefore usually non-HRMs were associated with this reluctance:

***“‘…Oh I'm a Doctor now, I know stuff,’ and with the pressure of, sort of, more, you know, people who are maybe, sort of, a little bit more senior than you thinking ‘what’s wrong with him… you don’t wanna [want to] always be seen to be in, you know, ‘what’s the dose of paracetamol?’…” (P2)***.

Moreover, both types of mistakes with non-HRMs were similar, with both having the reluctance of the junior doctors to ask seniors as a latent condition. Participant 23 described this when he prescribed the wrong dose of antibiotic:

***“Q***: ***So you say that you, now you're experienced you would ask, did you not ask in, when you first started?***

***A: I think it's sort of a bit of scare, scariness, I was, I'd probably go and just check in the BNF at that stage.” (P23)***.

Organisational documents led to knowledge based mistakes with HRMs only. Using trade names is considered to be an inappropriate prescribing habit, rather than an error. However, when trade names were used within organisational documents such as formularies, it can lead to errors as prescribers may not be prompted to remember the active ingredient or indeed know it in the first place. This occurred with an interviewee who prescribed a penicillin-based antibiotic combination for a patient who was allergic to penicillin, the hospital formulary guidelines used the trade name (Timentin) and that misguided the prescriber:

***“… I think it's because on, on this particular document that we have as, as a reference it uses, it uses a trade name as, rather than a generic name… they [trade names] can be confusing and conflicting and not really tell you what it is if you're in a rush.” (P2)***.

## Discussion

This study is the first to explore and compare the causes of prescribing errors and violations with HRMs and non-HRMs. We found that violations were not linked to HRMs and were only reported in prescribing errors with non-HRMs. This finding could be explained by the heightened risk of harmful consequences with HRMs in comparison to non-HRMs which may lead prescribers to avoid violations with HRMs. Although violations are intentional deviations from safe practice or the written rules of the working place, the violations described in this study with non-HRMs were intended to ensure patient benefit and to overcome workplace complexity. This finding is similar to previous findings that most violations are well-intended.[41] As well, our results confirmed that time pressures, leading to prescribers being in a hurry, high workload and lack of resources could lead to violations.[42]

Missing information from prescriptions, i.e. incomplete prescriptions, is a quite common type of prescribing error, [10] but they were not described with non-HRMs in this study. It is possible that, because HRMs have a potentially serious impact on patient safety, errors with these drugs were remembered by the prescribers for longer compared to non-HRMs. Therefore, when prescribers were interviewed they could recall them more readily than errors with non-HRMs. Furthermore, prescriptions for controlled drugs have strict legal prescription writing requirements and dispensing will be delayed until they are correct. In summary, controlled drug prescriptions seemed more susceptible to prescribing errors in comparison to other medications, particularly due to missing information. Therefore, techniques for targeting this type of prescription could improve patient care and save the time of the healthcare team.

Communication errors, such as poor written or verbal communication, have been reported previously, regardless of medication class or medication classification, without considering HRMs specifically. [16, 19, 21, 22] Sometimes communication errors could be described outside the medical team only, i.e. with other services or departments, as observed by *Kopp et al*. [18] In our study, communication errors with HRMs were associated with communication failures with other teams and non-HRMs were associated with communication failures inside the medical team. It could be hypothesised that this could be related to the nature of non-HRMs as being less harmful or viewed as less important, which result in neglect from both juniors and seniors to communicate effectively. On the other hand, concerns about HRMs may have led the medical team to communicate freely without any barriers although further, empirical work would be required to confirm this.

Barriers to good communication that were found included a reluctance by junior doctors to ask seniors (or other healthcare professionals), which could lead to unsafe prescribing and even violations of prescribing rules. Reluctance to ask advice from others and its impact on the safety of prescribing process has been described before. [21, 22, 43] However, the finding that this is particularly associated with mistakes with non-HRMs is new. Poor prescribing with non-HRMs was potentially perceived as more acceptable than with HRMs, making junior doctors less likely to contact their seniors and deal with prescribing decisions themselves.

Seeking senior advice was a concern to the junior doctors which could negatively affect patient safety; in contrast, some senior doctors in other studies have described feeling discomfort if the juniors did not seek help.[43] Therefore, junior doctors should be encouraged to seek help from seniors regardless of the medication class and senior doctors should be encouraged to make themselves available, especially with non-HRMs. Moreover, health institutes should encourage an open culture of communication between staff regardless of the steep hierarchy to attain openness and a positive safety culture.

Medication safety initiatives could target HRMs. For example, the use of on-line teaching has been found to improve medical students’ ability to calculate doses[44] and the development of an eLearning package that focuses on improving calculations with HRMs might be an effective way of improving calculation performance. There should be greater pharmacist-led support for junior doctors[45], which could include guidance on completion of controlled-medication discharge prescriptions. Ensuring that organizational documents (i.e. hospital drug charts) are designed so as not to propagate prescribing errors.[46] Interventions should be interactive such as workshops and feedback on previous prescribing tasks to help doctors change their prescribing behaviour.[47, 48] Moreover, approaches could include appropriate information provision and support such as providing junior doctors with practical, easy to understand and easy to access guidelines and key people to contact such as drug information specialists.

Future research could focus on a specific period of the prescribing process such as at discharge or during the on-call period, as these were described as particularly difficult times to prescribe safely placing a huge burden on the prescriber. Some HRMs, such as controlled drugs, insulin and anticoagulants were described as more challenging to prescribe safely. Therefore, focusing our efforts on understanding the nature of prescribing errors during the on-call period with HRMs would be beneficial to developing solutions to ensure that prescribers, especially junior prescribers (who most commonly work out of hours), can prescribe safely and that patient harm is prevented. Moreover, targeting error reduction strategies at HRMs in particular is more beneficial, especially for hospitals with limited resources, as they are less in number and high in harm in comparison to non-HRMs.

### Strengths and limitations of the study

This is the first study to investigate thoroughly the causes of prescribing errors with HRMs and compare them with the causes of errors with non-HRMs. Although it used secondary data analysis, the data were collected in original studies that related closely to the current research question. Therefore, this current study represented a more focused analysis of the data, meaning that our findings can be regarded with validity and reliability.[31] Furthermore, the large number of interviews (59) included descriptions of many junior doctors’ prescribing errors with both HRMs and non-HRMs, resulting in data saturation. Indeed, the analysis of Set 3 did not reveal any new findings and merely confirmed the findings from the previous sets. Therefore, there was robust intra-comparison between HRMs and non-HRMs achieving the aims and objectives of this study.

Using secondary analysis of existing data [27] makes the most of already rigorously collected data by investigating new concepts which were not investigated or asked initially [27, 49, 50]. This avoids further data collection, which is the most costly and time consuming phase of the research study. [49, 50] HRMs were not the focus of the primary research yet this group of medication could compromise patient safety more than general medications. However, secondary analysis of data also has it disadvantages as the original studies were based on a different research questions. Because of this there can be shallow and less detailed descriptions of the phenomenon under study and the original studies were not designed to explicitly compare HRMs and non-HRMs and information about for example non-HRMs might not be described as fully as they are not as memorable as errors with HRMs. This was demonstrated in our study as some interviews (n = 12) and prescribing errors were excluded because they could not fully answer or they did not fit our research question. However, the quality and richness of the remaining data was sufficient for us to achieve the aim of this study.

Secondary analysis can also mean that there are problems due to the researcher’s unfamiliarity with the details of the original studies and this can sometimes lead to misinterpretation of the data. [27, 31, 51] However, two of the authors of this current study were also authors of the original three studies and possessed detailed knowledge of the primary data.

## Conclusion

The differences between HRMs and non-HRMs reflect the nature of the prescribed medication class and the attitude of the prescribers toward the prescribed medication. Prescribers’ confidence with non-HRMs and the reduced sense of risk could lead to breaking the rules or reluctance to seek help. HRM related errors were illustrative of the complexity of the prescribing task with HRMs or additional rules regarding HRM prescriptions (e.g. controlled medication). Furthermore, medication class had an influence on communication as non-HRMs were associated with different types of communication failures than HRMs. In conclusion, these findings highlighted some of the areas that could be targeted by future interventions to reduce prescribing errors and increase patient safety, with both HRMs and non-HRMs.
